# Aging in place in rural Northeast China: a mixed methods examination of the influence of social capital on mental well-being in middle and late adulthood

**DOI:** 10.3389/fpubh.2023.1261132

**Published:** 2024-01-05

**Authors:** Yunfei Gao, Jing Wu

**Affiliations:** ^1^Department of Sociology, School of Law, Changchun University of Science and Technology, Changchun, Jilin, China; ^2^Department of Sociology and Work Science, University of Gothenburg, Gothenburg, Sweden

**Keywords:** social support, social trust, mental well-being, poverty, aging in place, middle-aged and older people, rural China, mixed methods

## Abstract

The rural development strategy in contemporary China has evolved from focusing solely on “absolute poverty alleviation” to addressing multiple dimensions, including “targeting relative poverty and revitalizing the entire rural area.” Using a mixed-methods approach, our study aimed to examine whether and how social capital, particularly social support and social trust, influences the mental well-being of middle-aged and older people aging in place in a remote rural Northeast area of China, exploring three constructs: life purpose, self-actualization, and capability. Our quantitative findings revealed that higher levels of social support and social trust were positively related to higher levels of life purpose. Increased social support was positively related to increased life purpose through increased social trust. However, the associations between social support, social trust, and the constructs of self-actualization and capability were not substantiated after controlling for covariates. Our interview data illuminated how middle-aged and older people perceived the interplay between social support, social trust, and mental well-being.

## Introduction

Social capital has been an essential concept in social, political, and behavioral sciences for decades (1). Both quantitative and qualitative studies have prospered, exploring the relationship between various facets of social capital (i.e., social support, social trust, social participation, and social networks) and physical and mental health not only in Western societies (2–4) but also in low and middle-income countries (5, 6).

Over the past few decades, a rich body of research has paid much attention to the impact of social capital on the health and well-being of China’s rural populations, encompassing varied age demographics, among the general population including middle-aged and older age groups (6–8) or specifically among older people (9–15). A study by Wang et al. (7) elucidated the nuanced effects of interpersonal trust and mistrust on general health and mental health, revealing these impacts at the individual level are contingent upon the village context. It was observed that individual-level trust had enhanced positive effects in villages characterized by low trust levels and antagonism, whereas the positive impacts of individual-level mistrust were more noticeable in mixed-motive villages featuring both high trust and mistrust levels (7). In another nationwide study (9), drawing on data from the Chinese General Social Survey (CGSS), it was observed that bonding social capital, such as trust, was exclusively associated with physical health and well-being among older urban Chinese rather than their counterparts in rural areas. This study stressed the importance of the social and cultural context in evaluating the effects of social capital on health and well-being (9).

Most of these studies tended to have mainly focused on either self-reported general health and/or mental ill-health, for example, depression (14). While several studies, such as those by Yip et al. (6) and Wang et al. (15), have delved into the impact of social capital on the emotional aspect of mental well-being, specifically life satisfaction, they have predominantly focused on either the general rural population (6) or older people in rural areas with appreciable economic and social resources (15). However, there is a noticeable research gap regarding the functional aspect of mental well-being, such as growth and achievement, particularly among rural aging populations who are navigating through limited resources and impoverished neighborhoods. Moreover, given that previous studies have underscored the significant role of social context and environment, in shaping the impact of social capital on well-being (3, 5, 7), our study seeks to extend this dialog by specifically exploring the relationship between social capital and mental well-being in middle and late adulthood in poverty-stricken rural areas. In addition to social context, Moore and Kawachi (1) suggested that the research on social capital and health should take into consideration a mixed methods approach to gaining a better understanding of various strategies by which individuals and groups access social capital for health benefits. However, to our knowledge, there are scarce studies exploring the relationship between social capital and mental well-being by using a mixed methods approach. In this study, building on a mixed methods research design, the study aims to (1) examine social support and social trust and their interactive effect on mental well-being in middle-aged and older people; and (2) explore how middle-aged and older people in a remote, poverty-stricken rural area perceive the interconnection between social support, social trust and mental well-being.

### Background

This study was initiated under a province-funded social science research project entitled “*Research on Behavioral Public Policy in Poverty Alleviation in Jilin Province after 2020 − Stimulating the Endogenous Power of Poverty Alleviation with ‘Spiritual Poverty Alleviation*’” (2019–2022), rooted in L town, Wangqing County, Yanbian District, Jilin Province. With a once nationally recognized impoverished status, Wangqing County, hosting 21 villages in L town and a population exceeding 20,000, has substantial relevance for scrutinizing and monitoring the living conditions of its aging residents. The county government declared the achievement of poverty alleviation in April 2020, making a nationwide alleviation of absolute poverty and shifting the focus toward addressing relative poverty and, particularly, the ongoing challenge of spiritual poverty in rural areas of China. Prior research (12) argued that targeted poverty reduction should not merely prioritize material poverty but also spiritual poverty concerning labor resources amidst drastic structural changes in rural China’s factor endowments due to urbanization and industrial upgrading. It implies that the government needs to manage rural aging adults’ mental health issues. Our study therefore underscores the necessity for policies to uplift economic conditions and resources for mental health and well-being needs among aging populations.

Due to emigration, natural resources changes and modernization in villages of rural Northeast area (16), It is found that a huge number of the young labor force were out to work, and a large proportion of middle-aged and older people were left behind in the village and they mainly did farm work. Demographic composition in this rural area to some extent influences the structural social capital (social contacts and reciprocity) and cognitive social capital (social support and social trust) among the residents, especially among those in middle and late adulthood (5, 7). Therefore, this study seeks to explore social capital and mental well-being among aging adults in a remote rural area of Northeast China, aiming to fill an existent research gap concerning the relationship between social capital elements (i.e., social support and trust) and mental well-being in China’s remote rural areas. Our focus is driven by the recognized influence of social capital on health in various contexts and the pronounced importance of social networks in regions lacking formal social welfare systems (6).

### Conceptual framework of social capital

Social capital is defined as connections among individuals, such as social networks and the norms of reciprocity and trustworthiness that arise from public health and community development perspective (1, 17). The community here is a collective character generated via social networks, reciprocity, and trust among residents and it could be a neighborhood, a local area, a town or city, a state, or a nation (18). Social capital’s multifaceted nature can be compartmentalized into three distinct categories: bonding, bridging, and linking. The bonding social capital implies informal close ties and support with and from family, friends, and neighbors (17, 19), and the bridging social capital includes weaker or more distant ties to people with whom we spend less time and have less in common (17, 19). As the third and final type, linking social capital covers formal connections and trust with someone in power or authority, whereby individuals can get access to services and assistance from well-resourced associations and organizations (17, 19, 20).

Social capital can also be measured at both structural and cognitive levels. The structural social capital refers to the presence or absence of formal opportunity structures or activities through which people may develop social ties and build social networks (1). The quantity of social ties, the function of networks, and the subjective evaluation of individuals on their networks may be the key ingredients of the structural social capital (1, 21–23). The cognitive social capital focuses on individuals’ perceptions and actions of trust, reciprocity, and support in a socially beneficial way (1, 7, 24). Trust has been a central measure to research on cognitive social capital and health (1, 25).

From a social capital setting perspective, social capital can be understood within family, neighborhood, and workplace (1). Coleman (26) stressed that family social capital needs to include all members and their relationships and interactions even though the relationship between children(ren) and parents in a nuclear family has primarily been discussed. According to Carpiano (18), neighborhood social capital as social resources that are inherent and embedded within community networks needs more attention from academics to assess its effect on health and well-being. As such, our study navigates through the poverty-stricken rural areas in Northeast China, aiming to explore the various facets of social capital, such as social support and social trust, and ascertain its impact on mental well-being among aging populations.

#### Social support and social trust

Social support and social trust as dimensional terms of social capital have been found to be associated with mental well-being (4, 27, 28). Social support refers to a form of social capital that individuals can draw upon to cope with daily problems (18). The benefits provided by social support are generally restricted to people who are part of the network that generates their resources (18). Social support consists of emotional, instrumental, appraisal, and informational components that may impact on health and well-being through a variety of direct, mediating, and moderating pathways (29–31). In our study, social support will be categorized into formal social support from the state and the village governments and informal social support from the neighborhood, including family, relatives, friends, and neighbors.

Social trust is a subjective attitude of individuals, and it implies that individuals have a good understanding of others, and believe that they can develop smooth connections and cooperation with their surroundings (32, 33). According to Putnam (34), social trust can be divided into generalized trust and particularized trust. The former refers to individuals’ perceptions of the trustworthiness of the social environment in general and, the latter is the perception and action of trust in their close interpersonal relations derived from their familiar social networks and neighborhood. In our study, social trust will be categorized into trust in the village government of which formal social networks provide formal social support, and trust in the neighborhood of which informal social networks offer informal social support.

### Mental well-being

As a complex subjective state, mental well-being has not yet been defined so far (3). Mental well-being is a concept that refers to a set of psychological processes that promote positive outcomes in a person’s life and it encompasses dimensions of hedonic well-being (i.e., positive feelings, affect, emotions) and eudemonic well-being (i.e., positive functioning, mindset, and relationships) (35). Individuals’ mental well-being may be shaped and conditioned by the prevailing values and social-ecological conditions of the environment (36). According to Jahoda (37), six criteria for positive mental well-being are as follows: attitudes toward the self; degree of growth, development, and self-actualization; personality integration; autonomy; adequate perception of reality; and environmental capability. In our study, the facets of mental well-being will be constructed to explore the differential effect of social capital on different measures of mental well-being in middle-aged and older people in the social context of rural Northeast China.

### The influence of social support and social trust on mental well-being

According to a Finnish study by Forsman et al. (4) based on qualitative data materials and quantitative survey data, informal social contacts such as family members and life-long relationships between friends impact the experienced mental well-being among older people owing to shared life events, social support, mutual appreciation, and trust, as well as a sense of belonging through common social activities. Hu and Qian (38) examined and revealed the relationship between social support and mental well-being among older people in the United Kingdom and the United States. In rural China, mutual aid and social support among neighbors, friends and family are very common on the base of traditional culture (39). Even though modernization to some extent makes the intensity and pattern of social support change, this informal support and reciprocity among residents have long sustained the well-being and quality of life of those people who inhabit more isolated, agricultural, and geographically dispersed areas (39). We, therefore, expect that high levels of social support make for high levels of mental well-being.

*Hypothesis 1*: Middle-aged and older people who experienced higher levels of social support, both formal from the government and informal via social networks, were more likely to exhibit higher levels of mental well-being, after controlling for all covariates.

Social trust as a key component of social capital has been taken best advantage of in previous studies to measure the cognitive dimension of social capital (7, 9, 11). Thomas and Streib (40) claimed that government trust derived from (1) close familial and geographic closeness, and (2) the action, legitimation, guidance, social regulations and (3) the reciprocity between the government and citizens. Trust as one key indicator of social capital, was related to personal well-being (3, 41) and trust in neighbors and trust in people in general were positively associated with the mental well-being of individuals (3, 42). Lu’s study (32), involving 6,891 participants from rural China, demonstrated that middle-aged and older people aged 50–69 years old exhibited the highest level of interpersonal trust compared to their younger counterparts. One pertinent study concerning left-behind older people in rural China discerned associations between trust at the individual level (in family members, neighbors, friends, and strangers) and diminished depression levels (11). Likewise, a study by Nyqvist et al. (2) found positive correlations between experienced interpersonal trust and a sense of security and mental health among a general adult population in Finland. Wang et al. (7) concluded that trust at the individual level derived from interpersonal relations was associated with mental well-being in rural China. We, therefore, expect that a high level of trust is a key explanatory factor for the high prevalence of mental well-being.

*Hypothesis 2*: Middle-aged and older people who experienced higher levels of social trust, both formalized toward government and informally oriented toward social networks, were more likely to exhibit higher levels of mental well-being, after controlling for all covariates.

The study of Kääriäinen and Lehtonen (43) examined how social networking, informal social support and social trust are connected to the way in which the states participate in producing social security in Nordic countries based on their social democratic welfare model. They found that when informal social support prevails, social trust grows (43). Likewise, Forsman et al. (4) examined the influence of social capital on mental well-being among older people in Finland and concluded that mental well-being is affected by informal social contact with family members through mutual social support and perception of trust. Moreover, research within the Chinese context, such as Yip et al. (6), elucidated the interplay of trust (i.e., both trust in individuals and trust in institutions) and support (i.e., emotional, and practical support from family, friends, and neighbors) that positively impacts health and well-being of rural residents. We, therefore, expect that social trust may mediate the impact of social support on mental well-being.

*Hypothesis 3*: Social trust, in its formal and informal variants, mediates the relationship between social support, both formal and informal, and mental well-being, after controlling for all covariates.

**Figure 1:**
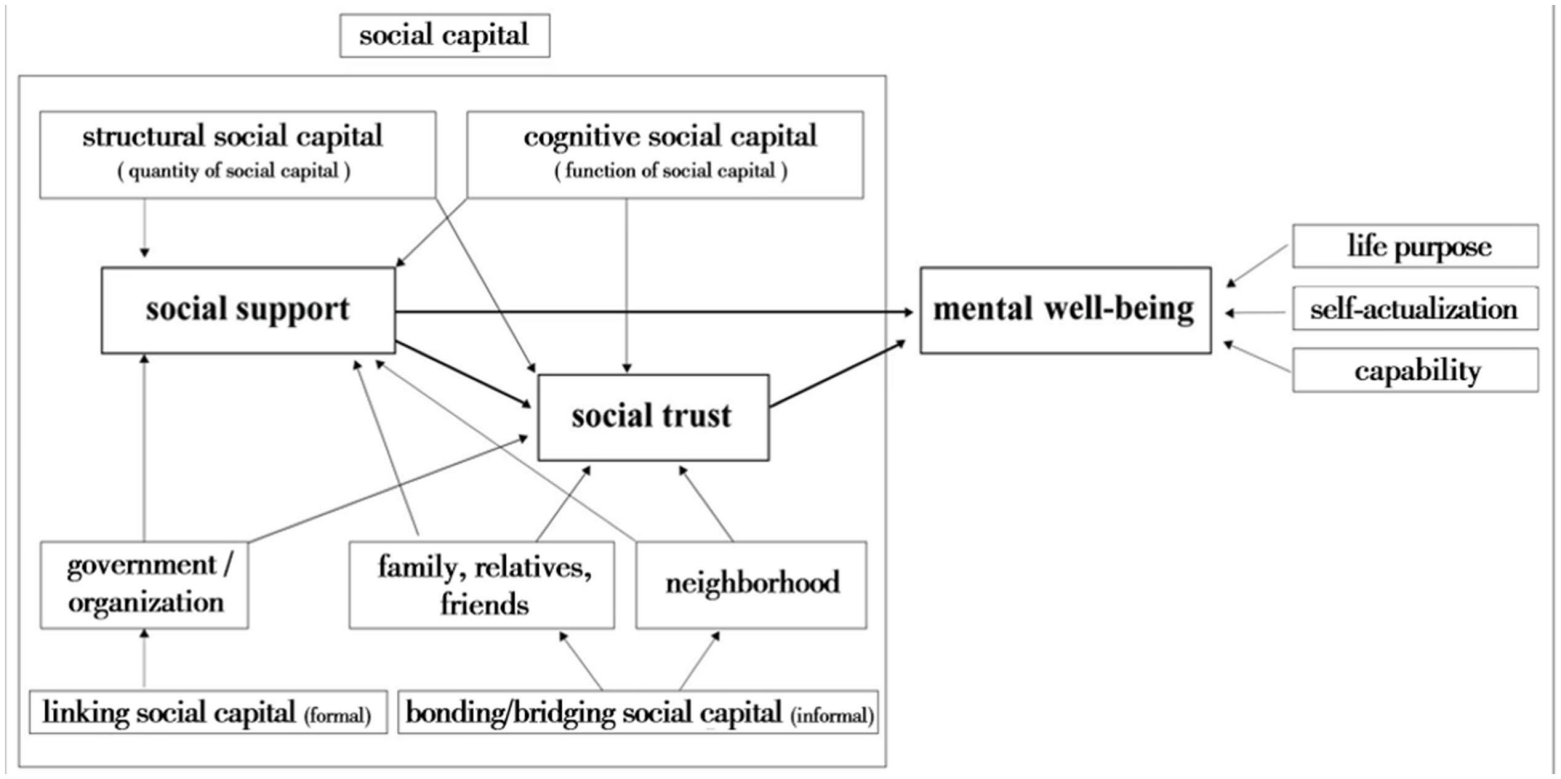
Conceptual model.

Based on the conceptual model (Figure 1), this mixed-methods study aims to examine the relationships between various facets of social capital (specifically social support and social trust) and mental well-being in aging populations.

## Materials and methods

### Survey sample

This mixed-methods study leveraged original data through a survey and in-depth interviews, originating from a research project focused on poverty alleviation in Jilin Province. The survey was conducted face to face and the interviews via telephone. Due to COVID-19, the investigation was conducted among inhabitants of several county villages from June 2021 to August 2022, despite project activation from August 2019 to August 2021. The pandemic and its aftermath affected research modalities, with a survey in July 2021 deploying face-to-face, in-home interviews. In total 352 people responded to the survey and 165 people were aged 50–83 among it. After the face-to-face survey, phone interviews were implemented from 24th June 2022 to 2nd July 2022, due to recurrent pandemic conditions and early winter onset in the Yanbian area. Ten people among those 165 samples who were eligible for the study were selected based on age and interview willingness and invited to have an in-depth interview via telephone. Those middle-aged and older people with severe physical and mental illnesses and sociopsychological life conditions were not included in the interview. The interviews were conducted predominantly in the evenings to accommodate the farming busy season during the daytime, typically lasting between 20 and 40 min.

As shown in Table 1, among those 165 individuals aged 50–83, the mean age was 58.72, and 51.5% were females. 82.4% of respondents had up to middle school education level, and 49.1% of respondents had annual net income below 4,000 RMB. 30.3% of them reported poor health. In quantitative analysis, socioeconomic status (i.e., educational level and income), age, gender, and self-reported health were included as covariates in the following analysis.

**Table 1:** Characteristics of the study population, *n* (%).

Characteristics	n (%)
Age (50–83)	
Mean	58.72
SD	7.34
Gender	
Men	80 (48.5)
Women	85 (51.5)
Education	
Primary school	67 (40.6)
Middle school	69 (41.8)
High school	19 (11.5)
Vocational education	6 (3.6)
University education	4 (2.4)
*Per capita* annual net income	
Below 4,000 RMB	81 (49.1)
4,000–10,000 RMB	54 (32.7)
10,000–20,000 RMB	23 (13.9)
20,000–30,000 RMB	3 (1.8)
Above 30,000 RMB	4 (2.4)
Health	
Very good	14 (8.5)
Good	40 (24.2)
Fair	61 (37.0)
Poor	39 (23.6)
Very poor	11 (6.7)
*N*	165

### Dependent variable

The dependent variable was mental well-being. According to the criteria of mental well-being (37), eight questions with 5-item answers in the survey were used to assess the mental well-being of the respondents. The questions were (1) I lead an active life to achieve my life goals; (2) I recently have been working hard to achieve my life goals; (3) I pursue my life goals with confidence; (4) I have succeeded in making dreams come true since I made all efforts on it; (5) I endeavor to overcome obstacles; (6) I have a good harvest since I have competence with farming; (7) A good quality of life of mine is based on my own capability; (8) Capability is a key component of success. As shown in Table 2, the answers for these above questions using the five-point Likert scale ranged from 1 = “Strongly Disagree” to 5 = “Strongly Agree.” KMO and Bartlett’s test of sphericity showed that the strength of the relationship was high and significant (KMO = 0.788, *χ*^2^ (28) = 431.622, *p* < 0.001); thus, exploratory factor analysis was suitable for the examination. Eight questions from the survey were therefore examined by using Principal Component Analysis, resulting in three factors explaining 72.011% of the variance. Thus, Factor 1 with an eigenvalue of 3.581 was named life purpose; Factor 2 with an eigenvalue of 1.121 was named self-actualization, and Factor 3 with an eigenvalue of 1.059 was named capability. The reliability of factor analysis was tested, and Cronbach’s Alpha was 0.809.

**Table 2:** Factor loadings of the eight manifest indicators on the three factors representing mental well-being.

Variable	Factor 1	Factor 2	Factor 3	Communalities
Life purpose (1)	0.841			0.777
Life purpose (2)	0.862			0.796
Life purpose (3)	0.838			0.750
Self-actualization (1)		0.691		0.695
Self-actualization (2)		0.794		0.782
Self-actualization (3)		0.774		0.663
Capability (1)			0.780	0.688
Capability (2)			0.875	0.610

Extraction method: Principal component analysis.

Rotation: Varimax with Kaiser Normalization.

Questions for life purpose (1*−*3) were (1) I lead an active life to achieve my life goals; (2) I recently have been working hard to achieve my life goals; (3) I pursue my life goals with confidence. Questions for self-actualization (1*−*3) were (1) I have succeeded in making dreams come true since I made all efforts on it; (2) I endeavor to overcome obstacles; (3) I have a good harvest since I have competence with farming. Questions for capability (1), (2) were (1) A good quality of life of mine is based on my own capability; (2) Capability is a key component of success.

### Independent variable

The independent variable was social support as one dimension of cognitive/bonding social capital (18, 29–31). Based on the questionnaire, four questions with 5-item answers were used to assess social support of the respondents. The questions were (1) My relatives and friends are there for me when I encounter some problem; (2) My family can give me essential help and support; (3) I can get emotional help and support from my family; (4) My relatives and friends will comfort me when I am in trouble. As shown in Table 3, the answers for these above questions using the five-point Likert scale ranged from 1= “Strongly Disagree” to 5 = “Strongly Agree”. KMO and Bartlett’s test of sphericity showed that the strength of the relationship was high and significant (KMO = 0.727, *χ*^2^ (6) = 186.603, *p* < 0.001); thus, exploratory factor analysis was suitable for the examination. Four questions from the survey were examined by using Principal Component Analysis, resulting in one factor explaining 61.885% of the variance. Thus, Factor 1 with an eigenvalue of 2.475 was named social support. The reliability of factor analysis was tested. Cronbach’s Alpha was 0.793.

**Table 3:** Factor loadings of the four manifest indicators on the one factor representing social support.

Variable	Factor 1	Communalities
Social support (1)	0.806	0.650
Social support (2)	0.780	0.609
Social support (3)	0.731	0.535
Social support (4)	0.826	0.682

Extraction method: Principal component analysis.

Rotation: Varimax with Kaiser Normalization.

Questions for social support (1*−*4) were (1) My relatives and friends are there for me when I encounter some problem; (2) My family can give me essential help and support; (3) I can get emotional help and support from my family; (4) My relatives and friends will comfort me when I am in trouble.

### Mediator variable

Mediator variable was social trust as one dimension of cognitive/linking social capital (7, 9, 11), specifically trust in village community. These questions with 5-item answers were selected from the survey to construct this mediator variable. The questions were (1) I am proud of my village committee when it has been praised by the local government; (2) My village community will have a better development; (3) I have a strong social connection and reciprocity with my village community. As shown in Table 4, the answers for these above questions using the five-point Likert scale ranged from 1 = “Strongly Disagree” to 5 = “Strongly Agree”. KMO and Bartlett's test of sphericity showed that the strength of the relationship was high and significant (KMO = 0.678, *χ*^2^ (3) = 114.809, *p* < 0.001); thus, exploratory factor analysis was suitable for the examination. Three questions from the survey were examined by using Principal Component Analysis, resulting in one factor explaining 66.661% of the variance. Thus, Factor 1 with an eigenvalue of 2.000 was named community trust. The reliability of factor analysis was tested. Cronbach’s Alpha was 0.737.

**Table 4:** Factor loadings of the three manifest indicators on the one factor representing social trust.

Variable	Factor 1	Communalities
Social trust (1)	0.797	0.635
Social trust (2)	0.852	0.726
Social trust (3)	0.799	0.639

Extraction method: Principal component analysis.

Rotation: Varimax with Kaiser Normalization.

Questions for social trust (1−3) were (1) I am proud of my village committee when it has been praised by the local government; (2) My village community will have a better development; (3) I have a strong social connection and reciprocity with my village community.

### Analytical approach

Utilizing an explanatory mixed-methods framework, this study adeptly intertwines quantitative and qualitative methodologies to dissect the intricate relationship between the mental well-being of middle-aged and older people in rural China and social capital, specifically social support and trust. Embarking first on a quantitative survey, followed by nuanced qualitative interviews (1), the two-phase design of the research ensures a thorough and robust exploration of the data (44). The initial quantitative phase sets the stage, to which the qualitative phase thoughtfully responds, offering a deeper dive into the preliminary findings (44). This methodological approach not only underscores any disparate results but also illuminates the multifaceted aspects of the phenomenon under study, facilitating a more comprehensive investigation into the elements that may have otherwise remained unexplored (45). Adhering to recommended practices, the qualitative design envisages engaging ten interview participants, ensuring a balanced and in-depth exploration of experiences and perspectives (46).

In the quantitative part, first, we tested bivariate correlations between social support, social trust, and mental well-being to evaluate the linearity assumption of multiple regression, and weak correlations may suggest a variable’s incompatibility with the model. Second, we utilized multiple linear regression modeling to test the effect of social support and social trust on mental well-being after controlling for the covariates. Last, we tested the substantial mediating effect of social trust on the relationship between social support and mental well-being. All statistical analyses were performed using IBM SPSS Statistics for Mac, Version 28.9 (Armonk, NY: IBM Corp).

To complement our quantitative findings, the qualitative analysis was conducted to explore how respondents perceived their mental well-being, and social capital, in particular, social trust and social support. We selected ten people from the respondents who participated in the quantitative survey and conducted in-depth interviews with them via telephone. As shown in Table 5, among these ten interviewees, there were five males and five females. Six interviewees were aged 50–59 and four were aged 60–66. NVivo package software was utilized to analyze data. Thematic analysis served as the analytical strategy for interpreting qualitative results, allowing for a rich and nuanced understanding of the research question (47). This analysis encompassed the identification of themes, where relevance is based on the research focus, question, context, and theoretical framework within which the research is conducted (48). Themes were identified using top-down coding, where we imposed a coding scheme on a body of non-numerical material (48).

**Table 5:** Statistics of interviewees.

ID	Interviewee	Gender	Age
1	F1	Female	54
2	F2	Female	51
3	F3	Female	50
4	F4	Female	52
5	F5	Male	51
6	F6	Male	60
7	F7	Male	66
8	F8	Female	57
9	F9	Male	60
10	F10	Male	61

## Results

### Quantitative results

#### Bivariate correlation

As shown in Table 6, social support as an independent variable had a significant and positive relationship with each factor construct of mental well-being as dependent variable. Moreover, social support had a significant and positive relationship with social trust. Social trust as mediator variable had significant and positive relationship with mental well-being as dependent variable.

**Table 6:** Bivariate analysis between social support, social trust, and mental well-being.

Variable	Life purpose	Self-actualization	Capability	Social support	Social trust
Social support	0.399**	0.290**	0.190*	1	0.530**
Social trust	0.387**	0.197*	0.197*		1

#### Regression analysis

Three-step hierarchical linear regression by enter method was utilized to test whether social support predicts mental well-being through social trust (Table 7). Social support and mental well-being factors were entered at stage one. Covariates including age, gender, educational level, income, and self-reported health were entered into the model at stage two. In stage three, social trust as a mediator variable was entered into the model. The models were significant for the first construct of mental well-being − life purpose. For self-actualization, models 1 and 2 were significant but not for model 3 when social trust entered the model. For capability, only model 1 was significant. When controlling for covariates and entering social trust variable in the later stages, the models were not significant. In the models of life purpose, it is obvious that when social trust as mediator variable entered the model, the coefficients of social support varied accordingly, which implies that social trust to some extent has a mediating effect. This offers a foundation for further conducting mediation analysis in the next step.

**Table 7:** Linear regression models for social support, social trust, and mental well-being with and without covariates (*N* = 138).

		Life purpose^c^	Self-actualization^d^	Capability^e^
Model 1^a^	Social support	0.399*** [0.247–0.562]	0.290*** [0.131–0.463]	0.190* [0.024–0.363]
Model 2^b^	Social support	0.413*** [0.268–0.570]	0.238** [0.086–0.401]	0.238** [0.072–0.412]
Model 3^b^	Social support	0.236** [0.062–0.416]	0.192* [0.005–0.389]	0.213* [0.009–0.425]
	Social trust	0.316*** [0.140–0.501]	0.081 [−0.113–0.279]	0.044 [−0.168–0.257]

a Not adjusted for covariates.

b Adjusted for age, gender, marital status, education, self-reported health.

c The model summary is significant in model 1 to 3.

d The model summary is significant in mode 1 to 2.

e The model summary is significant in model 1.

*p < 0.05, **p < 0.01, ***p < 0.001.

#### Mediation analysis

**Figure 2:**
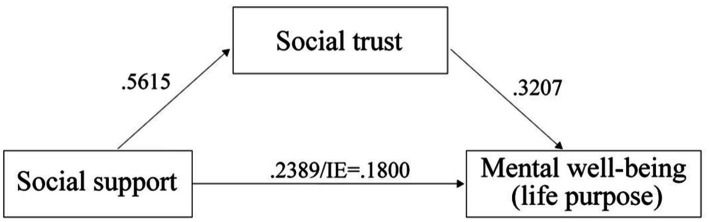
The mediating effect of social trust on the relationship between social support and mental well-being (life purpose).

Mediation analysis was applied with PROCESS macro to test the substantial mediating effect of social trust on the relationship between social support and mental well-being. We tested the mediating effect of social trust on the relationship between social support and three dimensions of mental well-being, separately. The meditating effect of social trust only held true for the relationship between social support and life purpose after controlling for all covariates, not for the relationship between either social support and self-actualization or social support and capability. Figure 2 incorporated the mediating effect of social trust on the relationship between social support and one construct of mental well-being − life purpose. It is shown that the path (direct effect) from social support to social trust was positively and statistically significant (*b* = 0.5615, s.e. = 0.0703, *p* < 0.001). The path (direct effect) from social support to life purpose was positive and significant (*b* = 0.2389, s.e. = 0.0895, *p* < 0.01), indicating that persons who receive higher social support are more likely to express an intention of life purpose than those scoring lower on the measure. The direct effect of social trust on life purpose was positive and significant (*b* = 0.3207, s.e. = 0.0913, *p* < 0.001), indicating that persons scoring higher on social trust are more likely to express an intention of life purpose than those scoring lower on the measure. The indirect effect (IE = 0.1800) is statistically significant: 95% CI = (0.0211, 0.2955), indicating that a higher level of social support has a positive effect on a higher intention of life purpose through a higher level of social trust.

### Qualitative results

In the quantitative segment, the attention was predominantly directed toward exploring social trust through a formal format, while social support was scrutinized more intensively within an informal framework. To expand the measurements of both social support and trust, the qualitative segment incorporated questions addressing support and trust in both formal and informal aspects. This approach ensured a comprehensive analysis aligned with the conceptual model, thoroughly exploring bonding, bridging, and linking forms of social capital.

#### High levels of social support

In the quantitative part, the respondents in the survey reported that family, relatives, neighbors, and friends provide them with social support in general. In qualitative interviews, we obtained more specific information in detail in terms of the support type. Among the ten interviewees, the primary support from their family members consisted of emotional support and daily interactions. However, some interviewees also noted that their families occasionally provided financial and material assistance.

*“We received financial support mostly from our older son. Our two children are out of town. They do not live with us. So we have contact and communication through WeChat.” (F1, 54 years old).*

*“I have a very good relationship with my son. He always helps me when I am farming.” (F2, 51 years old).*

*“I have two daughters. They can cook and clean up the house when they come back home. Both of them are very good at doing laundry.” (F3, 50 years old).*

*“I had contact with my children through WeChat and mobile.” (F7, 66 years old).*

*“(My) children live too far away from us. They cannot help us with farming, but they give financial help to us. We have emotional communication.” (F10, 61 years old).*

The emotional support of family members to the respondents was mainly reflected in the high frequency of communication. Some of their children lived far away from them so they communicated with children via telephone and social media applications (i.e., WeChat) etc.; for those interviewees whose children lived in the village close to them, their communication was mainly through frequent door-to-door visits. In addition, some interviewees helped to take care of their grandchildren. In rural areas of China, individuals often become grandparents earlier in life. By the time they reach 55, over 80% of those in their middle adulthood have already become grandparents (49). Through this caring activity, the interviewees communicated with their children more frequently, and grandparenting to some extent relieved negative emotions and improved the mental well-being of the interviewees (50).

#### High levels of social trust

Social trust mainly comes from two levels: trust in the village government and trust in the neighborhood. The respondents’ high trust in the village government was mainly because the village-level government is the most grass-roots organization in the countryside. The members of the village committee were responsible for not only communicating the principles and policies of the higher-level government to the village residents but also responsible for implementing various policies. Public opinion was gathered and sent out to the higher-level government, and this smooth communication is the basis of the high trust in the village government among village residents. Meanwhile, the implementation of various measures to benefit people in the national rural revitalization strategy has brought improvements to the living environment and material life of the village residents and enhanced their trust in the village government.

*“I trust the village government. For my later life in the future, I hope the village government can help me.” (F1, 54 years old).*

*“I trust the village government, the central government, and the state.” (F2, 51 years old).*

*“My husband has diabetes and I myself also went to the hospital for a health check. Life is good, (because) we get good benefits from the state…we have hope about the future.” (F4, 52 years old).*

*“Our government is trustworthy. What we hope to improve is that the state can boost policy to decrease the gap between the poor and the rich.” (F5, 51 years old).*

The interviewees, hailing from the studied county—previously designated as a national poverty-stricken county—experienced firsthand the initial era of national and regional anti-poverty strategies. Throughout this phase, the state funneled resources for poverty alleviation and rural development to poverty-afflicted counties via local governments at a macro level, thereby enhancing the economic status of the entire region (16). Under these circumstances, several interviewees stressed that it was the state and the village government that made for village residents’ good quality of life. Nowadays anti-poverty strategy in China entered its second era. Resources from the state were also transferred directly to rural households at a micro level. Consequently, it is a crucial mission yet a big challenge for the local villages to recognize who the targeted poor group is (16). It could be the potential reason why interviewee F5 expected that the national policy could decrease economic inequality between the poor and the rich.

#### The linkage of social support and social trust

Neighborhood trust is mainly manifested as a high-trust relationship between neighbors based on reciprocity. According to the interview results, the neighborhood relationship was very close, and this close relationship to some extent supplemented the relationship with family and in turn gave additional support and benefit to village residents, whereby trust in the neighborhood among them could be maintained and strengthened and as a result, the mental well-being could be improved.

*“(Neighborhood relations) very good, distant relatives are not as good as close neighbors. My neighbors help me feed and take care of pigs, chickens, and dogs at home when I am outside of home. If the neighbors cannot do farm work, I will help them. One of my neighbors had her legs broken, and all of us went to help her. I believe my help positively affects the financial situation and living standards of my neighbors.” (F1, 54 years old).*

*“I am very talkative and enthusiastic, so I tell you, for example, if today I urgently need money, I can ask my neighbors for help. They can immediately lend (transfer) money to my personal account* via *WeChat. You see, this is our good relationship with each other.” (F2, 51 years old).*

The interviewees’ perception of trust in their neighbors was intertwined with their actions demonstrating trust. According to Moore and Kawachi (1), it is difficult for individuals to engage in reciprocity exchanges or collective action without the perception of trust. In rural China, a majority of aging adults, as well as middle-aged individuals over 50, primarily engage in agricultural activities (51). Within this context, the interviewees F1 and F2 reported that they assisted their neighbors based on a high level of mutual trust. In return, the neighbors performed reciprocal behaviors, such as helping with agricultural work.

#### High levels of mental well-being dependent on high levels of social support and social trust

Even though the interviewees were not satisfied with their current financial situation and perceived that they had low socioeconomic status when compared with other groups, they admitted that their current living conditions have been exceedingly improved than before, which makes them feel satisfied with life in general and look forward to their economic prospects in the future. The interviewees reported that their satisfaction with life depended on a high level of trust both in the state and village committee (offering formal social support) and the neighborhood and family (offering informal social support).

*“Residing in the countryside (rural area), I do not have high expectations. I just want to live a stable life, and the (village) government and the state are both good, so that’s fine.” (F2, 51 years old).*

*“It’s okay now, life is good now.” (F6, 60 years old).*

*“I cannot say I’m satisfied or dissatisfied. Now the national policy is better, for example, we do not need to pay for the agricultural tax any longer.” (F7, 66 years old).*

The interviewee F7 stressed that life satisfaction was related to better policies from the state and the village committee. The interviewee further exemplified that the farmers do not need to pay for the agricultural tax anymore. The abolishment of agricultural taxes was in force in 2002 in China and following it, the central government launched a targeted poverty alleviation and rural revitalization strategy that lubricates the transfer of more benefits and resources to poverty-stricken rural areas (16). With high levels of formal social support and resources provided by the state, the village residents perceived high levels of mental well-being.

## Discussion

The rural development strategy in contemporary China has evolved from focusing solely on “absolute poverty alleviation” to addressing multiple dimensions, including “targeting relative poverty and revitalizing the entire rural area” (52). Based on it, our study aimed to quantitatively examine whether and how social capital, in particular, social support and social trust, influences the mental well-being of middle-aged and older people aging in place in a remote rural Northeast area of China. In addition, the study aimed to qualitatively explore how the respondents perceived the social support and social trust derived from their formal and informal social networks in the village community.

We hypothesized that high levels of social support and social trust are positively related to high levels of mental well-being and social trust might mediate the relationship between social support and mental well-being. Our results partially supported these hypotheses. In the quantitative part of our research, when considering various dimensions of mental well-being, our hypotheses received full support concerning life purpose. Namely, support and trust, and their interplay, impact life purpose. It is widely recognized that China’s rural areas uphold and perpetuate cultural traditions that encompass elements like ethics, obligations, trust, reciprocity, networking, human affection, sympathy, and solidarity (39). These elements not only foster internal resources but also establish a mutual assistance system in rural areas (53), forming a pivotal aspect of village residents’ social capital. This social capital presumptively influences their mental well-being (52), with our study highlighting a particular inclination toward life purpose, as evidenced in both quantitative and qualitative results. Moreover, our qualitative results underscored the critical role of village committees. Recognized as trustworthy agents providing formal social support to residents, these committees effectively link internal and external resources, enhancing the quality of life and mental well-being of aging adults. This aligns with past research affirming the relationship between social networks, social support, social trust, and the mental well-being of individuals in rural settings (54, 55). Furthermore, the impact of social support on individual well-being and quality of life has proven even more potent in extremely impoverished rural areas (54). Particularly, social trust, especially trust in the government, can alleviate the health and psychological vulnerability of rural residents facing poverty risk (55).

While Hypothesis 1 (positing that social support positively correlates with self-actualization) was supported, neither social support nor social trust, nor their interplay, was positively associated with self-actualization or capability, counter to what was anticipated based on the previous study (52). The quantitative part of the study did not affirm hypotheses in the realms of self-actualization or capability, potentially due to the measurements used for social support and trust. The former leaned more toward informal support from close relations, omitting formal support from the government, and the latter skewed toward civic trust based on familial and geographical closeness among residents, and reciprocal relationships with village government, omitting political trust measurements pertaining to legitimacy, actions, and governance (40). For example, research conducted in rural areas of Shandong Province (10) revealed that informal social support, like emotional and practical aid from family and friends, correlated strongly with physical health, while formal support, including expert advice and information, exhibited a stronger association with mental health and well-being. This study (10) underscored the importance of examining the impact of diverse forms of social support on health and well-being in the contexts of a rapidly aging rural population, insufficient pension support, and migration of working-age populations.

In terms of the study’s context, institutional trust that encompasses government and rural political trust represents both the public’s subjective evaluation and emotional closeness to governmental governance, and an embodiment of the interaction between them (56–58). The latter implies that the economic expectations of the residents in the village can be met, and the prestige of the local village government can be maintained (59). Moreover, trust in the community/neighborhood acts as a “moral” resource, facilitating collective action, reciprocity, and norm enforcement (1). Particularly in impoverished areas of rural China, such as the county studied in our research, where residents expect more formal social support and resources from the government, this trust becomes crucial. Some prior research even indicates that elevated political trust may foster a sense of security, stronger affiliations with legal systems, and subsequently, improved mental health and well-being (8). Future quantitative investigations may take into consideration measuring diverse forms of social support and social trust relating to mental well-being.

The qualitative portion of our study offered insights into potential barriers to self-actualization among rural middle-aged and older people, notably poor health, which many interviewees identified as a primary obstacle to achieving goals like financial improvement. Interviewees generally expressed an expectation for augmented resources and support from both village and central governments, with an emphasis on more generous healthcare compensation. Utilizing data from the China Family Panel Studies (CFPS) 2016 that encompasses 95% of mainland China’s population across 25 provinces/municipalities/autonomous regions, Liang et al.’s study (13) implies several policy implications to bolster health in rural aging populations of China, such as crafting rural organizations attentive to the localized needs and characteristics of the aging populations and allocating more public resources to underpin local activities to foster greater social capital accumulation among rural aging populations in China (13).

### Policy implications

Our study advanced the understanding of social capital among middle-aged and older people in rural Northeast areas, a topic that demands yet often lacks sufficient scholarly and policy attention. Our study showed the importance of social capital in bolstering mental well-being and further building endogenous development capacity among middle-aged and older people in these regions. From the initial anti-poverty program and strategy on targeted poverty alleviation to the up-to-date anti-poverty actions and performances inclined to rural revitalization and community development, the essence of related policies is the transformation from material poverty to spiritual poverty, in particular lack of endogenous development capacity at the community level (52) and at the individual level (16).

Gao (60) noted that China’s first rural social relief program was the Five-Guarantee Household System for the poorest older people, which implies that social assistance programs are for poor and ‘deserving’ people instead of a preventive form of social insurance that covers all groups of citizens based on a social rights perspective. The development of total welfare for aging adults is, therefore, inadequate, and incompatible with social needs and economic conditions (60). Both quantitative and qualitative results from our study reveal that middle-aged and older people with poor material situations have high trust and expectations in the state and village committee in terms of formal social support and resources. Furthermore, the interviewees expressed a desire for additional resources and benefits from the state and the village governments and sought more emotional connections from family and neighborhood. Previous studies have also shown that the current policies need to scrutinize whether their interventions and strategies effectively incorporate social capital at the community and individual levels into policy design and evaluation (1, 13, 61). Therefore, the village governments should formulate strategies to create age-friendly communities that support people aging in place in rural areas and leverage internal and external resources to help rural residents build their own social capital and sustain high levels of mental well-being.

### Methodological limitations

The findings of the study should be considered in light of certain limitations regarding the measurement tools employed. A small sample of middle-aged and older people was recruited in the villages where the project team had worked, which may not authentically represent the typical aging population in rural China. Additionally, the quantitative part had a cross-sectional design so the causal relationship between social capital and mental well-being cannot be completely ensured. Third, the quantitative data were collected through self-reported questionnaires and therefore some recall biases were inevitable. Moreover, all interviews were conducted via telephone with durations ranging from 20 to 40 min, constrained by the COVID-19 pandemic and the busy farming season during the study, which could potentially impact the quality of the results. Last, the survey omitted certain key socio-demographic characteristics, such as marital status and household size, from the questionnaire, possibly leading to an absence of crucial information regarding social capital, especially concerning the quantity of social networks and social capital. Despite these limitations, the study sheds light on valuable insights into the relationships between social capital and mental well-being among aging populations in rural, remote, and impoverished areas of China.

## Conclusion

The strength of our study lies in the utilization of a mixed methods approach, which further highlights the importance of both formal and informal social support and social trust for the mental well-being of middle-aged and older people aging in place in the remote rural Northeast areas of China. The study advances the current understanding of different types of social capital, including formal social support from the village government and the state, emotional closeness with family members (especially children and/or grandchildren), and instrumental reciprocity and trust with neighbors. This suggests that special attention should be paid to the macro-level social welfare system and its application to the rural aging populations in poverty-stricken rural areas. In addition, our study provides further evidence to support the idea that social support and social trust are important to facilitate middle-aged and older people to improve their endogenous motivation and capacity and in turn improve their well-being and quality of life. Future research should take into consideration how middle-aged and older people as crucial actors in poverty elimination can optimally utilize resources provided by the government via formal social support and social trust, actively engage in poverty alleviation activities, and ultimately make their needs met and their voices heard.

## Data availability statement

The raw data supporting the conclusions of this article will be made available by the authors, without undue reservation.

## Ethics statement

This study was approved by the Institutional Review Board (IRB) of the School of Law, Changchun University of Science and Technology and all research subjects gave their informed consent for inclusion before they participated in the study.

## Author contributions

YG: Conceptualization, Formal analysis, Methodology, Software, Supervision, Validation, Visualization, Writing – original draft, Writing – review & editing, Data curation, Funding acquisition, Investigation, Project administration, Resources. JW: Conceptualization, Formal analysis, Methodology, Software, Supervision, Validation, Visualization, Writing – original draft, Writing – review & editing.

## References

[1] Moore S Kawachi I. Twenty years of social capital and health research: a glossary. J Epidemiol Community Health. (2017) 71:513–7. doi: 10.1136/jech-2016-208313 28087811

[2] Nyqvist F Finnäs F Jakobsson G Koskinen S. The effect of social capital on health: the case of two language groups in Finland. Health Place. (2008) 14:347–60. doi: 10.1016/j.healthplace.2007.09.001, PMID: 17950023

[3] Nyqvist F Page B Pellfold T Forsman A Wahlbeck K. Structural and cognitive aspects of social capital and all-cause mortality: a meta-analysis of cohort studies. Soc Indic Res. (2014) 116:545–66. doi: 10.1007/s11205-013-0288-9

[4] Forsman AK Herberts C Nyqvist F Wahlbeck K Schierenbeck I. Understanding the role of social capital for mental wellbeing among older adults. Ageing Soc. (2013) 33:804–25. doi: 10.1017/S0144686X12000256

[5] De Silva MJ Huttly SRA Harpham T Kenward MG. Social capital and mental health: a comparative analysis of four low income countries. Soc Sci Med. (2007) 64:5–20. doi: 10.1016/j.socscimed.2006.08.044, PMID: 17045716

[6] Yip W Subramanian SV Mitchell AD Lee DTS Wang J Kawachi I. Does social capital enhance health and well-being? Evidence from rural China. Soc Sci Med. (2007) 64:35–49. doi: 10.1016/j.socscimed.2006.08.027, PMID: 17029692

[7] Wang H Schlesinger M Wang H Hsiao W. The flip-side of social capital: the distinctive influences of trust and mistrust on health in rural China. Soc Sci Med. (2009) 68:133–42. doi: 10.1016/j.socscimed.2008.09.038 18986744

[8] Lin X Lu R Guo L Liu B. Social capital and mental health in rural and urban China: a composite hypothesis approach. Int J Environ Res Public Health. (2019) 16:665. doi: 10.3390/ijerph16040665 30823510 PMC6406475

[9] Norstrand J Xu Q. Social capital and health outcomes among older adults in China: the urban–rural dimension. Gerontologist. (2012) 52:325–34. doi: 10.1093/geront/gnr072, PMID: 21746837

[10] Sun X Liu K Webber M Shi L. Individual social capital and health-related quality of life among older rural Chinese. Ageing Soc. (2017) 37:221–42. doi: 10.1017/S0144686X15001099

[11] Ke Y Jiang J Chen Y. Social capital and the health of left-behind older adults in rural China: a cross-sectional study. BMJ Open. (2019) 9:e030804. doi: 10.1136/bmjopen-2019-030804, PMID: 31772090 PMC6886947

[12] Wang Y Wang L Wu H Zhu Y Shi X. Targeted poverty reduction under new structure: a perspective from mental health of older adults in rural China. China Agric Econ Rev. (2019) 11:555–66. doi: 10.1108/CAER-12-2018-0243

[13] Liang H Yue Z Liu E Xiang N. How does social capital affect individual health among the elderly in rural China? Mediating effect analysis of physical exercise and positive attitude. PLoS One. (2020) 15:e0231318. doi: 10.1371/journal.pone.0231318, PMID: 32716935 PMC7384663

[14] Lu N Wu B Pei Y Peng C. Social capital, perceived neighborhood environment, and depressive symptoms among older adults in rural China: the role of self-rated health. Int Psychogeriatr. (2022) 34:691–01. doi: 10.1017/S1041610221000958 34365988

[15] Wang X Wang P Wang P Cao M Xu X. Relationships among mental health, social capital and life satisfaction in rural senior older adults: a structural equation model. BMC Geriatr. (2022) 22:73. doi: 10.1186/s12877-022-02761-w, PMID: 35073854 PMC8785491

[16] He S Wang W. Social resources transfer program under China’s targeted poverty alleviation strategy: rural social structure and local politics. J Contemp China. (2022) 32:686–03. doi: 10.1080/10670564.2022.2109844

[17] Putnam R. Bowling alone: the collapse and revival of American Community. New York: Touchstone (2001).

[18] Carpiano RM. Toward a neighborhood resource-based theory of social capital for health: can Bourdieu and sociology help? Soc Sci Med. (2006) 62:165–75. doi: 10.1016/j.socscimed.2005.05.020, PMID: 15992978

[19] Kyne D Aldrich DP. Capturing bonding, bridging, and linking social capital through publicly available data. Risk Hazards Crisis Public Policy. (2020) 11:61–86. doi: 10.1002/rhc3.12183

[20] Aldrich DP. Building Resilience: Social Capital in Post-disaster Recovery. Chicago: The university of Chicago press (2012). 248 p.

[21] Eriksson M Ng N. Changes in access to structural social capital and its influence on self-rated health over time for middle-aged men and women: a longitudinal study from northern Sweden. Soc Sci Med. (2015) 130:250–8. doi: 10.1016/j.socscimed.2015.02.029 25734610

[22] Kahn RL Toni A. Convoys over the life course: attachment, roles, and social support In: Balters P Brim O, editors. Life-Span Development and Behavior. New York: New York Academic Press (1980). 254–83.

[23] Wu J Hasselgren C Zettergren A Zetterberg H Blennow K Skoog I. The impact of social networks and APOE ε4 on dementia among older adults: tests of possible interactions. Aging Ment Health. (2020) 24:395–04. doi: 10.1080/13607863.2018.1531368 30587010

[24] Abbott S Freeth D. Social capital and health: starting to make sense of the role of generalized trust and reciprocity. J Health Psychology. (2008) 13:874–83. doi: 10.1177/1359105308095060 18809638

[25] Carpiano RM Fitterer LM. Questions of trust in health research on social capital: what aspects of personal network social capital do they measure? Soc Sci Med. (2014) 116:225–34. doi: 10.1016/j.socscimed.2014.03.017, PMID: 24721251

[26] Coleman J. Social capital in the creation of human capital. Am J Sociol. (1988) 94:S95–S120. doi: 10.1086/228943

[27] Almedom A. Social capital and mental health: an interdisciplinary review of primary evidence. Soc Sci Med. (2005) 61:943–64. doi: 10.1016/j.socscimed.2004.12.025 15955397

[28] De Silva MJ McKenzie K Harpham T Huttly SRA. Social capital and mental illness: a systematic review. J Epidemiol Community Health. (2005) 59:619–27. doi: 10.1136/jech.2004.029678, PMID: 16020636 PMC1733100

[29] Henriques A Silva S Severo M Fraga S Barros H. Socioeconomic position and quality of life among older people: the mediating role of social support. Prev Med. (2020) 135:106073. doi: 10.1016/j.ypmed.2020.106073 32243939

[30] Son H Cho HJ Cho S Ryu J Kim S. The moderating effect of social support between loneliness and depression: differences between the young-old and the old-old. Int J Environ Res Public Health. (2022) 19:2322. doi: 10.3390/ijerph19042322 35206508 PMC8871923

[31] Wu J Kasearu K Värnik A Tooding LM Trommsdorff G. Associations between quality of relationships and life satisfaction of older mothers in Estonia, Germany Russia and China. Ageing Soc. (2016) 36:1272–94. doi: 10.1017/S0144686X15000355

[32] Liu C. An analysis of the influence of social trust in rural residents. Statis Manag. (2019) 12:8–11. doi: 10.16722/j.issn.1674-537x.2019.12.002

[33] Zheng H. Reconstruction of social trust: a perspective of “new rural construction” from the “home construction action plan” of Wuhan. Learn Prac. (2006) 3:79–82+1. doi: 10.19624/j.cnki.cn42-1005/c.2006.03.014

[34] Putnam R. Bowling alone: America’s declining social capital. J Democr. (1995) 6:65–78. doi: 10.1353/jod.1995.0002

[35] Koushede V Lasgaard M Hinrichsen C Meilstrup C Nielsen L Rayce SB. Measuring mental well-being in Denmark: validation of the original and short version of the Warwick-Edinburgh mental well-being scale (WEMWBS and SWEMWBS) and cross-cultural comparison across four European settings. Psychiatry Res. (2019) 271:502–9. doi: 10.1016/j.psychres.2018.12.003 30551082

[36] Chan MF Isnis I Chu CW Ling C Ling SY. Development and validation of a mental wellbeing scale in Singapore. Psychology. (2013) 4:592–06. doi: 10.4236/psych.2013.47085

[37] Jahoda M. Current Concepts of Positive Mental Health. New York: Joint Commission on Mental Illness and Health (1958).

[38] Hu Y Qian Y. COVID-19, inter-household contact and mental well-being among older adults in the US and the UK. Front Sociol. (2021) 6:714626. doi: 10.3389/fsoc.2021.714626 34381838 PMC8350320

[39] Bian G Liu N. Rural mutual aid tradition and its change and rural social welfare construction. Future Dev. (2010) 31:20–24+19.

[40] Thomas JC Streib G. The new face of government: citizen-initiated contacts in the era of E-government. J Public Adm Res Theory. (2003) 13:83–102. doi: 10.1093/JOPART/MUG010

[41] Sum S Mathews MR Pourghasem M Hughes I. Internet technology and social capital: how the internet affects seniors’ social capital and wellbeing. J Comp Med Comm. (2008) 14:202–20. doi: 10.1111/j.1083-6101.2008.01437.x

[42] Theurer K Wister A. Altruistic behaviour and social capital as predictors of well-being among older Canadians. Ageing Soc. (2010) 30:157–81. doi: 10.1017/S0144686X09008848

[43] Kääriäinen J Lehtonen H. The variety of social capital in welfare state regimes: a comparative study of 21 countries. Eur Soc. (2006) 8:27–57. doi: 10.1080/14616690500491399

[44] Creswell JW Plano Clark VL. Designing and Conducting Mixed Methods Research. Thousand Oaks: Sage (2007). 274 p.

[45] Teddlie C Tashakkori A. Overview of contemporary issues in mixed methods research In: Teddlie C Tashakkori A, editors. SAGE Handbook of Mixed Methods in Social and Behavioral Research. London: Sage Publications (2010). 1–42.

[46] Collins KMT. Advanced sampling designs in mixed research: current practices and emerging trends in the social and behavioral sciences In: Teddlie C Tashakkori A, editors. SAGE Handbook of Mixed Methods in Social and Behavioral Research. London: Sage Publications (2010)

[47] Braun V Clarke V. Using thematic analysis in psychology. Qual Res Psychol. (2006) 3:77–01. doi: 10.1191/1478088706qp063oa

[48] Bergman MM. Hermeneutic content analysis: textual and audiovisual analyses within a mixed methods framework In: Teddlie C Tashakkori A, editors. SAGE Handbook of Mixed Methods in Social and Behavioral Research. London: Sage Publications (2010)

[49] Zhang J Emery T Dykstra P. Grandparenthood in China and Western Europe: an analysis of CHARLS and SHARE. Adv Life Course Res. (2020) 45:100257. doi: 10.1016/j.alcr.2018.11.003 36698270

[50] Zhang J Fokkema T Arpino B. Loneliness among Chinese older adults: the role of grandparenthood and grandparental childcare by gender. J Fam Issues. (2021) 43:3078–99. doi: 10.1177/0192513X211041992

[51] Cheng X Fang Y Zeng Y. How long can Chinese women work after retirement based on health level: evidence from the CHARLS. Front Public Health. (2023, 2023) 11, 11:987362. doi: 10.3389/fpubh.2023.987362, PMID: 36923039 PMC10009266

[52] Wen J Liu Y. Toward a new endogenous era: The endogenous development dilemma and strategies of rural revitalization. Guizhou Soc Sci. (2022) 389:142–9. doi: 10.13713/j.cnki.cssci.2022.05.015

[53] Zhang W Zhang Z. Resources, participation and identification: The endogenous development logic and path selection of rural revitalization. Soc Sci. (2018) 11:75–85. doi: 10.13644/j.cnki.cn31-1112.2018.11.008

[54] Ye C Luo L. Social capital, poverty alleviation policy and household welfare of the poor: Hierarchical linear analysis based on the rural survey data of Guizhou Province. Finan Sci. (2011) 7:100–9.

[55] Zhang Y. Social capital and the health vulnerability of poor farmers: Inhibition or promotion? Based on the data from China family panel studies survey. Xinjiang State Farms Econ. (2022) 6:1–9.

[56] Dong L Sun S. A study on political trust in rural areas of China: Based on the empirical analysis of 6 townships in Y City S Province. J Fujian Admin Instit. (2016) 6:55–61. doi: 10.19357/j.cnki.35-1295/d.2016.06.008

[57] Li Y. The conceptual connotation, forming factors and political function of government trust. Jinyang J. (2007) 3:22–6. doi: 10.16392/j.cnki.14-1057/c.2007.03.003

[58] Zhang L. Political trust: the way to maintain stability in rural areas. J Shanxi Youth Manag Cadre Coll. (2012) 4:46–8.

[59] Li G Meng Y. The change and enlightenment of rural political trust relationship in the 70 years since the founding of New China. J Shandong Agric Eng Coll. (2020) 9:1–6. doi: 10.15948/j.cnki.37-1500/s.2020.09.001

[60] Gao J. Characteristics of China’s residual welfare system for elderly people. China J Soc Work. (2014) 7:288–04. doi: 10.1080/17525098.2014.962759

[61] Productivity Commission. Social Capital: Reviewing the Concept and Its Policy Implications. Melbourne, Australia: Commission Research Paper (2003). 100 p.

